# ‘Menopause affects us all . . .’: menopause transition experiences of female ambulance staff from a UK ambulance service

**DOI:** 10.29045/14784726.2021.12.6.3.41

**Published:** 2021-12-01

**Authors:** Larissa Stella Prothero, Theresa Foster, Debra Winterson

**Affiliations:** East of England Ambulance Service NHS Trust ORCID iD: https://orcid.org/0000-0002-5440-8429; East of England Ambulance Service NHS Trust; East of England Ambulance Service NHS Trust

**Keywords:** ambulance, female, menopause

## Abstract

**Background:**

There is limited research regarding the menopause transition in the emergency services; however, all women will experience this life phase, which can have a significant impact on personal well-being, workplace attendance and performance. The aim of this survey was to explore personal and work impacts of the menopause for all female staff in the ambulance setting.

**Methods:**

A purpose-designed, 20-question survey, based on the Menopause Rating Scale and British Menopause Survey, was developed to understand menopausal symptoms and their impact on female staff in one UK ambulance service. Disseminated during 1–31 July 2019, it resulted in a convenience sample of 522 responses, which were analysed using descriptive statistics and thematic approaches.

**Results:**

Typically, respondents were either pre-menopausal or peri-menopausal, with approximately a third being menopausal or post-menopausal. Over half worked in emergency operational delivery, and typically worked shifts or unsocial hours. For those who had experienced menopause symptoms, the most commonly reported were tiredness or low energy levels, difficulty sleeping (including insomnia) and mood changes (including anxiety or depression). Symptoms impacted respondents’ well-being, work and home life. Most had not expected the symptoms they experienced. The majority of respondents did not feel supported at work, with lack of menopausal symptom awareness and personal impact, working times and patterns, and sense of embarrassment of most concern. Other issues included lack of managerial and peer support, inadequate working environment and uniform, lack of dignity and choice, and no dedicated menopause policy.

**Conclusions:**

It is understood that this is the first survey to explore female ambulance staff menopause experiences. The impact of menopausal symptoms can be significant. Menopause awareness in this ambulance service is lacking and there is clear scope for initiatives for improved staff support and well-being. Further research is warranted to explore how best to support ambulance staff with the menopause transition.

## Introduction

The menopause (also known as the menopause transition) is being increasingly recognised as an important workplace issue. Menopausal symptoms can have a significant impact on female health and well-being and are known to affect work attendance and performance. Two recent national reports from the British Menopause Society have highlighted the impacts of the menopause (British Menopause Society, 2017a, 2017b), and in early 2020, the NHS Staff Council’s Health, Safety and Wellbeing Partnership Group published their first ‘Menopause at Work’ guidance, to support NHS organisations to improve the way they address the menopause and work, and offer guidance for line managers and staff (NHS Confederation, 2020).

Demographic analysis has shown that approximately 40% of the UK ambulance and paramedic workforce is female (Health and Care Professions Council, 2019; NHS Digital, 2019). Many will be aged 40 years or more (Health and Care Professions Council, 2019; NHS Employers, 2019), so likely to experience the menopause transition during their working lives. Typically, the menopause (i.e. cessation of menstrual periods) occurs at an average of 51 years of age and 75% of women experience menopausal symptoms (NHS, 2018). Those affected can find it difficult to function effectively and their working conditions exacerbate their symptoms. In addition, up to 10% of women can experience early menopause or premature ovarian insufficiency (both can be associated with typical menopausal symptoms). Furthermore, trans, non-binary and intersex people can also experience the menopause, and may face additional challenges and have differing support needs.

For UK Police and Fire Service female staff, the impact of the menopause has been recognised and support resources have been produced (Davies et al., 2018; Fire Brigades Union, 2016; National Police Chiefs’ Council Menopause Action Group, 2019; Watkins et al., 2019); however, only one NHS Ambulance Trust has produced guidance for its staff (East Midlands Ambulance Service NHS Trust, 2019). Consequently, the aim of this survey was to explore the impact of the menopause transition on women working within one UK ambulance Trust, to inform the need of menopause-related well-being support.

## Methods

A purpose-designed, online 20-question survey (available on request) was devised for a service evaluation, based on the Menopause Rating Scale (Heinemann et al., 2003; with permission) and British Menopause Survey (British Menopause Society, 2017a, 2017b). The questions covered topics such as length of work for the ambulance service, function of work, working patterns, driving requirements, age of respondents, stage of menopause, symptoms experienced and their severity, expectation and effect of symptoms, need for time off work and how supported women felt in and outside of work for their symptoms. The survey was advertised to all staff via an electronic newsletter and an email across the East of England Ambulance Service NHS Trust from 1 to 31 July 2019, to obtain a convenience sample. This approach was adopted as it was considered pragmatic for this setting. Survey completion was voluntary and anonymous, and informed consent was obtained prior to completion. No personally identifiable information was collected, and all responses were securely stored electronically (password protected).

All data analysis was completed using Microsoft Excel. Descriptive statistical techniques were used to analyse responses. The final survey question was a free-text option for additional comments to support any responses given. These responses were thematically analysed using an inductive approach, grouping comments with the same meaning together into themes (Braun & Clarke, 2006). LSP and TF independently reviewed all comments, generated initial codes and identified emerging themes based on the coding. They then reviewed and refined these themes together to agree meaningful study themes.

## Results

### Respondent demographics and employment

A total of 522 surveys were received (completion rate: 100%) and analysed, which at the time of the survey represented more than a fifth (22%) of all service female staff (n = 2364). Most respondents were less than 44 years (44%; n = 230), with 43% (n = 222) being 45–54 years, and 13% (n = 70) being 55 years or more. When asked how long they had worked for the ambulance service, most stated 10–19 years (39%; n = 204), then less than five years (28%; n = 146), 5–9 years (18%; n = 93) and 20 years or more (15%; n = 78).

All key service areas were represented: emergency operational delivery (58%; n = 301), support roles (17%; n = 89), ambulance operations centre roles (16%; n = 85), scheduled patient transport (4%; n = 19) and resilience and specialist operations (2%; n = 10). Three quarters (75%; n = 391) of respondents worked shifts or unsocial hours, with the remainder working regular office hours (25%; n = 132). In addition, for almost two-thirds of respondents, driving was a key part of their role (63%; n = 327).

### Menopause transition: symptoms and impact

**Figure 1:**
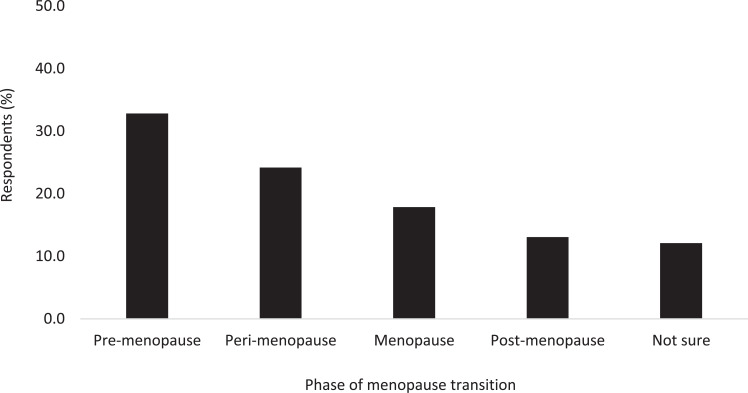
Figure 1. Prevalence of menopause transition phases within the survey cohort.

Almost a quarter of respondents reported to be peri-menopausal (24%; n = 126); under a third had reached the menopause or were post-menopausal (31%; n = 161); a third had not started the menopause transition, that is, were pre-menopausal (33%; n = 171); and the remainder reported to be unsure which phase they were experiencing (12%; n = 63: see Figure 1).

Of respondents in the menopause transition, approximately two-thirds experienced menopausal symptoms (64%; n = 336), which were numerous and varied in nature (see Table 1). Focusing on those rated moderate or severe, symptom frequency in descending order was: tiredness or low energy levels; difficulty sleeping, including insomnia; mood changes, including anxiety or depression; joint stiffness, aches or pains; hot flushes and/or night sweats; weight changes; headaches or migraines; period pattern changes; gastric problems; vaginal dryness, pain or itching; palpitations and skin changes; recurrent urinary tract infections; dry mouth; dental problems; hair loss; and altered taste. The majority of respondents (61%; n = 205) reported they had not expected the symptoms they experienced, and more than half had tried something to reduce or prevent their symptoms (59%; n = 198), including consulting a healthcare professional (51%; n = 171).

**Table 1.** Respondent menopause symptoms and severity. Prevalence is expressed as % (n); severity is described as none, mild, moderate or severe.

Symptom	Symptom severity			
	None	Mild	Moderate	Severe
**Period pattern changes**	26.3% (88)	14.1% (47)	16.8% (56)	14.1% (47)
**Vaginal dryness, pain or itching**	41.3% (139)	17.3% (58)	11.3% (38)	5.4% (18)
**Recurrent urinary tract infections**	56.3% (189)	9.1% (31)	5.3% (18)	5.5% (19)
**Difficulty sleeping (including insomnia)**	10.9% (37)	13.8% (46)	27.8% (93)	25.4% (85)
**Tiredness or low energy levels**	6.0% (20)	13.8% (46)	32.0% (108)	26.3% (88)
**Headaches or migraines**	20.7% (70)	25.6% (86)	23.0% (77)	9.0% (30)
**Mood changes (including anxiety or depression)**	10.1% (34)	19.8% (67)	31.1% (104)	17.1% (57)
**Problems with memory or concentration**	10.7% (36)	24.0% (81)	28.1% (94)	15.2% (51)
**Hot flushes (short, sudden feelings of heat, usually in the face, neck and chest, which can make your skin red and sweaty)**	19.9% (67)	17.0% (57)	20.3% (68)	20.1% (68)
**Night sweats (hot flushes that occur at night)**	19.0% (64)	18.1% (61)	20.0% (67)	20.0% (67)
**Palpitations**	37.6% (126)	24.6% (83)	12.4% (42)	2.7% (9)
**Weight changes**	18.6% (62)	22.1% (74)	28.0% (94)	8.5% (29)
**Joint stiffness, aches and pains**	11.4% (38)	23.4% (79)	29.2% (98)	13.7% (46)
**Gastric problems**	32.2% (108)	20.4% (69)	19.0% (64)	5.8% (19)
**Dry mouth**	50.7% (170)	17.2% (58)	7.4% (25)	1.9% (6)
**Altered taste**	55.4% (186)	16.0% (54)	4.3% (14)	1.4% (5)
**Dental problems**	54.9% (184)	13.6% (46)	6.2% (21)	2.7% (9)
**Skin changes**	36.4% (122)	25.5% (86)	13.2% (44)	2.3% (8)
**Hair loss**	51.1% (172)	17.3% (58)	8.2% (28)	0.8% (3)
**Other**	63.0% (212)	3.9% (13)	3.5% (12)	1.4% (5)

**Figure 2:**
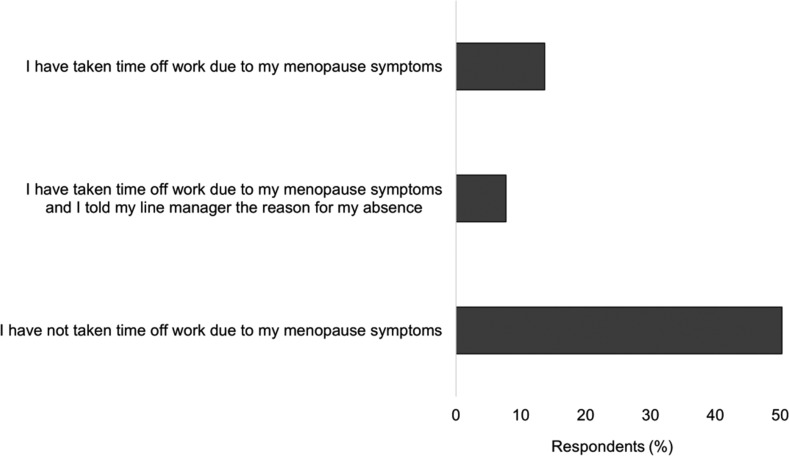
Figure 2. Respondents’ need for time off work due to menopausal symptoms.

Respondents were also asked whether they had needed to take time off work due to their menopause symptoms. Approximately a fifth (21%; n = 71) of those experiencing symptoms had needed time off work, and only about half of these women had told their line manager the real reason for their absence (54%; n = 38: see Figure 2).

**Figure 3:**
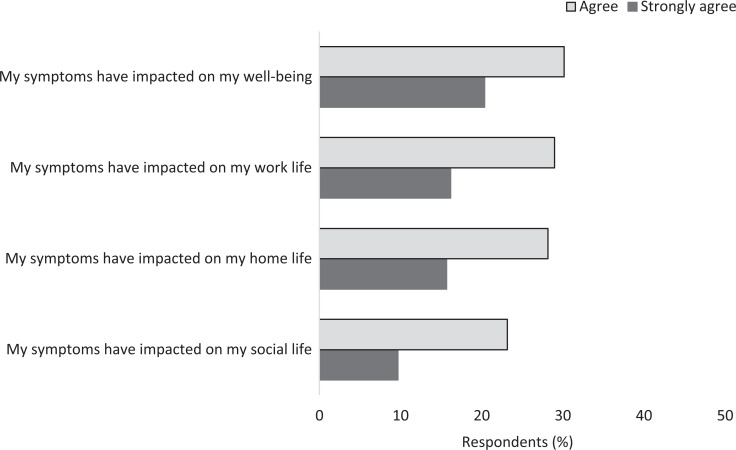
Figure 3. The impacts of menopausal symptoms for respondents.

**Figure 4:**
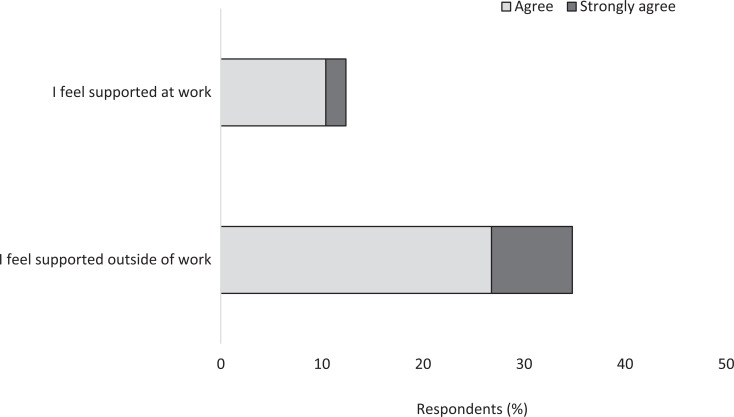
Figure 4. Level of support felt during the menopause transition in and outside of work.

Menopausal symptom impact could be significant, that is, respondents reported that their symptoms affected their personal well-being (51%; n = 171), work life (45%; n = 151), home life (44%; n = 148) and social life (33%; n = 111): see Figure 3. When asked how supported they felt with regard to their menopause symptoms, over a third (35%; n = 183) of respondents felt supported outside of work, while only 12% (n = 63) felt supported at work (see Figure 4).

### Respondents’ supporting comments

More than a quarter (27%; n = 142) of respondents provided free-text comments, the content of which was thematically analysed and revealed 10 broad themes, the overarching theme being lack of menopause awareness and symptom impact.

#### Awareness and preparedness for menopause transition symptoms

Respondents indicated a lack of awareness of the range and potential severity of menopause transition symptoms, and subsequently did not feel prepared for this phase:

I wasn’t prepared for the amount of anxiety I would feel and the stress it would cause me. (P2)

I would like more information, so I am prepared for going into the menopause . . . (P91)

Some of the symptoms I have I didn’t realise, until this survey, that migraines which is my main sickness problem, was a symptom of the menopause. (P108)

General awareness-raising throughout the service, in particular for management roles and male understanding, was mentioned frequently:

More understanding in management with less embarrassment to talk about these things. (P7)

I’ve found that men in the workplace don’t understand how the menopause makes you feel at all. (P75)

I do feel that the menopause is not understood by colleagues, not just the male ones but younger women who have not experienced this. (P111)

A better understanding of the menopause by everyone and how it can effect everyday life including work. (P344)

#### Impact of the menopause transition

This theme captured comments relating to personal impacts of the menopause transition, which included work performance and career choice:

Sleep deprivation due to night sweats, etc. can have a big impact. (P177)

I am really surprised at the foggy head and not remembering things quickly. Unable to sleep properly during the day for a night shift. (P295)

I think the biggest things that have impacted me so far are migraine headaches, being unable to get good sleep and erratic, sometimes very heavy periods. (P171)

The menopause in my experience has huge impacts on an individual’s life at work and at home, and the symptoms initially are not attributed to it. (P178)

There have been many times I have thought of resigning my job because of the impact of my peri-menopause symptoms. (P16)

#### Impact of working times and patterns

Comments within this theme involved difficulties surrounding shift-working, in particular night shifts, and the potential benefit of flexible working opportunities for menopause transition staff:

The opportunity for flexible working to combat the tiredness / poor sleep. (P78)

Alternative shift patterns to help reduce fatigue and be able to have more rest days between shifts especially after nights. (P358)

I have already changed my hours and felt I couldn’t work full time due to the symptoms I have. (P447)

Having structure would help, regular shifts and knowing when night shifts are going to be helps, relief is horrible. (P473)

#### Embarrassment surrounding menopause transition

Respondents also provided comments regarding feeling embarrassed about their experience of the menopause transition, and symptoms not being taken seriously by others:

It’s a bit of a reoccurring joke that if the older woman complains she’s hot she’s having a flush. (P31)

. . . Breakdown the embarrassment factor that people feel about a natural process. (P90)

Allow road crews to use facilities more frequently without having to explain why you need to every time – it’s embarrassing. (P138)

If I was to take time off work because of my symptoms I would probably be laughed at by management. (P435)

I am embarrassed by the symptoms I experience and not having such a good memory as I used to, it is almost like having a disability. (P445)

#### Managerial support

Comments in this theme were divided between what management support was perceived as helpful and unhelpful by respondents:

Great that its being addressed. What’s important to me is that I know I have an understanding Manager. (P62)

More sympathetic management would be helpful. Most women would rather not be going through menopause and having to take time off. (P160)

No manager was remotely interested in my menopause. I was not supported just laughed at. (P271)

Having a male line manager is difficult. (P294)

I have now been put on an action plan at work [for my symptoms] so no support from work. (P364)

#### Impact of working environment

Staff physical working environments and uniform provision were also of concern for respondents (predominantly relating to temperature management):

Fans in offices for women and also to be able to sit by open window and be able to take short break more often. (P15)

. . . I sit on the ambulance for my breaks with the air con on. (P198)

Some [personal protective equipment] is not menopause friendly, even in the cold, we are prone to hot flashes & need lighter options. (P237)

Lighter clothing options as the uniform is very hot to wear in summer. (P321)

#### Need for dignity

This theme captured some of the personal challenges faced by frontline operational ambulance staff while managing their menopausal symptoms:

Access to spare uniform for female road staff should you have an ‘accident’ as periods can be much heavier. (P37)

It’s often very difficult to manage symptoms whilst out on the road – headaches, super heavy/unreliable periods and tiredness/aching/exhaustion are often treated as excuses for laziness which is definitely not the case. (P197)

#### Support network importance

In this theme, comments encompassed the importance of talking about the menopause, and the perceived benefit of having a service support group or information champion:

. . . I think we should provide some literature for all female staff about the menopause and advise them of possible symptoms, helpful resources, and a place to signpost them to enabl[e] them to talk to someone. (P187)

This was talked at an NHSI session and it’s great to see the Trust taking some positive steps. There should be a network that could form part of [Service women’s support network]. (P206)

Perhaps create an in-house check for women starting or suffering with their menopause. (P269)

#### Sickness and menopause policy

The penultimate theme included negative comments related to the current sickness policy, and the perceived importance of a dedicated menopause policy:

A one-size fits all sickness policy does not support different groups of individuals such as those women suffering menopause symptoms. (P136)

The Trust should be able to think outside the box in relation to depression & low mood elements – it’s not as clear cut / black & white that you are either sick or not. (P143)

Was dealt with no differently to someone being off with a cold or back pain under the normal sickness policy. There was no understanding of the symptoms or allowances made. (P195)

#### Lack of choice

The final theme included comments around perceived difficulties in supporting women during the menopause transition, and lack of choice regarding seeking help:

It is what it is just got to get on with it. (P66)

Honestly wouldn’t know what to suggest as symptoms vary so much, how would you support someone with intermittent memory and concentration problems? (P401)

I did not expect any difference in treatment inside or outside work as it is a natural part of life. (P284)

## Discussion

It is understood that this is the first survey to explore the experiences and impact of the menopause transition in female ambulance staff. Responses were received from women experiencing the peri-menopause, menopause and post-menopause, as well as those who were yet to start the menopause transition. Respondents highlighted the complex array of menopausal symptoms they experienced, which were often not expected and could significantly impact both work and home lives. The three most challenging symptoms reported were tiredness or low energy levels, difficulty sleeping (including insomnia) and mood changes (including anxiety or depression). Such symptoms are generally typical of women working across a range of work settings, including the police and fire emergency services (Beck et al., 2020; Davies et al., 2018; Griffiths et al., 2013; Watkins et al., 2019).

Despite recent public awareness-raising activities in the UK, the menopause transition appears to remain a taboo subject in the workplace (Beck et al., 2020; Hardy et al., 2019). As this survey has shown, female ambulance staff can be reluctant to discuss the menopause with their peers and service managers, instead choosing to manage their symptoms privately, persisting with work and not seeking professional health advice. Lack of communication can contribute to the sense of lacking support. There is a tendency for service line management not to be informed of the real reason for work absence, and this has been observed in other work settings (Beck et al., 2020). Reasons for non-disclosure are known to be complex but have been reported to include: the menopause being a private issue not to be talked about at work; disclosure resulting in negative perceptions by colleagues; and an individual’s work abilities being questioned. As highlighted by some respondents’ comments, women can experience difficulties and embarrassment when discussing the menopause with male colleagues (Beck et al., 2020; Watkins et al., 2019).

It was beyond the scope of this service evaluation to identify and prioritise the activities needed to meet the needs of female ambulance staff experiencing the menopause transition. However, as identified in the responses, measures to address menopause awareness, physical work environment (in particular, temperature management and access to appropriate uniform and personal protective equipment), improved menopause-related communication skills and behaviours, and supportive policies and practices are common needs of menopausal women (Hardy et al., 2017). It is understood that other emergency services, in particular the police, are already providing menopausal support for their staff, via national menopause guidance (National Police Chiefs’ Council Menopause Action Group, 2019), awareness campaigns, provision of educational resources and ‘relief kits’ in cars and stations, and it would appear that provision of similar resources for female ambulance staff would likely be appreciated. The National Ambulance Diversity Forum (supporting the Association of Ambulance Chief Executives) has already been informed of this survey’s findings as it sought feedback ahead of a national uniform procurement arrangement. Furthermore, regardless of future in-depth research, this ambulance service is developing a dedicated menopause transition policy and guidance.

Several limitations of this evaluation are acknowledged: while a large number of staff participated in this survey, it is recognised that this is a single service evaluation so the generalisability of the findings may be limited; the purpose-designed survey used has yet to be formally validated, although it is based on the validated Menopause Rating Scale and British Menopause Survey; not all closed questions were mandatory, resulting in variable question completeness rates (n = 19; mean = 99%; range = 90–100%); as an anonymous survey, multiple entries from a single respondent could not be identified; and it was beyond the scope of the survey to explore issues for those identifying as trans, non-binary and intersex people; however, they may be captured by a proposed larger scale research study in this area.

## Conclusion

All women will experience the menopause transition and will work through and beyond this life phase. Our survey findings have shown that the physical and mental health impacts for female ambulance staff can be considerable. There is a clear need for improved menopause awareness across the ambulance service, with appropriate menopause-related resources to support all staff. These may assist with not only improving well-being, but also the retention of female staff through the menopause during their working journey.

(The findings described here were initially presented at the 999 EMS Research Forum 2020 (Foster et al., 2020)).

## Author contributions

The study concept was devised by TF. Both LSP and TF have contributed equally to the survey design, data analysis and reporting of findings. DW has provided oversight regarding well-being in the workplace for data analysis and interpretation. SQUIRE guidelines and the CHERRIES checklist provided frameworks for the preparation of this manuscript (Eysenbach, 2004; Ogrinc et al., 2016). TF acts as the guarantor for this article.

## Conflict of interest

LP is a member of the editorial board of the *BPJ*.

## Ethics

The Health Research Authority (HRA) (http://www.hra-decisiontools.org.uk/research/) did not deem the study to be research, therefore HRA approval was not sought. The East of England Ambulance Service NHS Trust Clinical Best Practice Group approved this study (reference: MIDWEST).

## Funding

None.
